# Sexuality in the light of awareness of approaching mortality

**DOI:** 10.1017/S1478951525100965

**Published:** 2025-11-14

**Authors:** Livia Kislev, Lena Kurtz-Almog, Pdut Bier, Rina Dikhel, Michal Avidan, Alexander Waller

**Affiliations:** Rehabilition Department, Israel Cancer Association, Giavatyaim, Israel

**Keywords:** Cancer, concerns, end of life, intimacy, sexuality

## Abstract

**Objectives:**

This study examined the changes in intimacy and sexuality amongst cancer patients at the end of life, including those in the final stage, and the distress they raised while experiencing those changes.

**Methods:**

A phenomenological qualitative study, based on interviews with 35 dying cancer patients. The results were analyzed by Constant Comparison Analysis method.

**Results:**

Some of the dying patients reported absence of essential change in their sexual needs and ability. while others reported about changes. The changes caused seven various forms of distress to the majority of patients, for example grief due to diminution of sexuality, impact on the partner due to lack of sex and distress resulting from consciousness of the end of life. A minority did not experience distress from the sexuality changes. About a third of the interviewees were interested in sexual counseling during their dying period, and about 80% considered it important for the palliative care team to raise the issue of sexuality.

**Conclusions:**

End of life patients and even those in final stages may have needs related to their intimate and sexual life. As long as the person breathes, even towards death, he can continue to live, even in the intimate aspect, so the palliative team has an important role in answering the specific and complex needs related to sexuality at the end of life. Recommendations were formulated specifically based on this research, for professional intervention regarding sexuality at the end of life, by a PASSION model.

## Introduction

According to the World Association for Sexual Health (WAS), sexuality is a fundamental aspect of human life, encompassing sex, intimacy, identity and gender roles, sexual orientation, eroticism, pleasure, and reproduction (Ford et al. 2022). However, most existing studies in this area focus on cancer survivors but not on individuals nearing the end of life (Goetsch et al. 2015; Den Ouden et al. 2019; Abbott-Anderson et al. 2020; Di Mattei et al. 2021), early-stage cancer patients (Jackson et al. 2016; Yesilbalkan and Gokcol 2017; La Spina 2024), or patients with chronic illnesses (Kelemen et al. 2019).

Research on sexuality in the context of terminal illness, particularly among cancer patients approaching death, remains limited. Few studies address this subject in any depth, and even fewer are based on empirical data (Taylor 2014; Wang et al. 2018). From a palliative care perspective, Hordern (2015) emphasizes that sexuality can reduce stress and anxiety, alleviate pain and insomnia, aid in muscle relaxation, offer distraction from symptoms, and foster emotional connection between partners. Additional studies have pointed to the release of oxytocin during sexual activity as a source of comfort, tranquility, and increased pain tolerance (IsHak et al. 2011). Despite these noted benefits, sexuality remains an understudied aspect of palliative care, particularly regarding patients’ subjective experiences and emotional responses to changes in their sexual lives during their terminal stage.

One of the rare quantitative studies on this topic, involving 65 patients in palliative care, found a sharp decline in sexual activity and satisfaction. Specifically, 66.6% reported engaging in penetrative sex prior to diagnosis, compared to only 7.6% during the palliative stage. Similarly, while 83% had previously experienced high sexual satisfaction, only 12.1% reported such satisfaction during the palliative phase. Nonetheless, nearly half of the participants reported an ongoing desire for physical and emotional intimacy with their partners (Vitrano et al. 2011).

Surviving partners have described a range of expressions of love and connection. Some abstaining entirely from sex, others maintaining physical closeness through caresses, and some continuing to engage in active sexual relationships as a means of comfort, expression of grief, or emotional release (Gianotten 2007).

The limited attention given to this topic may partly stem from what scholars have called “desexualization” – a phenomenon in which caregivers implicitly regard sexuality as irrelevant or inappropriate at the end of life. This reluctance to address sexuality may be rooted in a “double taboo” surrounding both sex and death (Wallach et al. 2023). Healthcare providers often report avoiding conversations about sexuality with terminal patients due to feelings of discomfort, embarrassment, perceived irrelevance, lack of training, or limited knowledge regarding sexuality in palliative contexts (Cagle and Bolte 2009; Leung et al. 2016; Benoot et al. 2018; Wallach et al. 2023). Some professionals have even questioned the appropriateness of raising the issue in palliative settings (Hjalmarsson and Lindroth 2020). Yet previous research suggests that many terminally ill patients would welcome the opportunity to discuss changes in their intimate lives with their medical team (Lemieux et al. 2004; Flynn et al. 2012).

This gap in literature – alongside healthcare professionals’ hesitancy to engage with the topic – highlights the need for more qualitative studies that center patients’ experiences and unmet needs related to intimacy and sexuality during the dying process.

## Research objectives

The study offers a novel contribution through in-depth interviews with terminal patients including those in the final stages of life regarding the changes they experience in their sexuality and the associated emotional impacts. It also investigates the degree of interest among these patients in receiving sexual counseling, and their perspectives on whether healthcare providers should address the issue proactively. The findings challenge common misconceptions about sexuality at the end of life and provide practical insights for improving patient care.

## Methods

### Study design

This study utilized a qualitative phenomenological design aimed at capturing the subjective experiences of terminal cancer patients without relying on prior assumptions. The decision to use in-depth interviews was grounded in ethical and methodological considerations, given the personal and sensitive nature of the topics involved – death, intimacy, and sexuality. Such issues often require a nuanced and empathetic approach to elicit authentic responses (Morse 2012).

The phenomenological approach enabled the research team to explore patients’ experiences in depth, facilitating rich, detailed insights into how sexuality and intimacy are perceived and experienced in the context of impending death.

### Participants

A total of 35 terminal cancer patients participated in the study. Of these, 18 were treated through the Israel Cancer Association’s Palliative Home Care Unit and 17 were hospitalized in an oncology ward in a central Israeli hospital.

Inclusion criteria were as follows:

- A prognosis of less than six months to live (as defined by Steinberg and Sprung 2007)
- Age 18 or older
- Fluency in either Hebrew or Russian

Participants’ demographic and clinical characteristics are presented in Table 1.

**Table 1.** Characteristics of research participants (*N* = 35)

Characteristic		*N*	%
Age range			
	18–40	4	11.4
	41–50	8	22.8
	51–60	7	20
	61–70	9	25.7
	71–80	7	20
**Gender**			
	Women	15	42.8
	Men	20	57.2
**Personal status**			
	First marriage	23	65.71
	Second marriage	5	14.28
	Divorced, in a relationship	2	5.72
	Single, in a relationship	3	8.57
	Single with no relationship	2	5.72
**Type of cancer**			
	Pancreatic	5	14.28
	Colorectal	6	17.14
	Prostate	4	11.42
	Lung	3	8.57
	Other: Gastric (2), Ovarian (2), Melanoma (2), Head & Neck (2), Kidney (2), Sarcoma (1), Brain (1), Chest (1), Liver (1), Leukemia (1), Thyroid (1), Unknown origin (1)	17	48.57
	**Number of interviewees**	**35**	

## Data collection

Data were collected using a semi-structured interview guide developed by the research team. The guide included the following:

- Part I: Demographic and clinical questions (16 items on background, 6 on illness-related information).
- Part II: Open-ended questions regarding patients’ experiences on changes in intimacy and sexuality, the distress associated with those changes, engagement in self-pleasure, and their thoughts on whether sexuality should be discussed by the medical team. The questions were designed based on the study aims, gaps in existing literature, and the teams’ clinical experience.

## Ethical considerations

Ethical approval was obtained from the Helsinki Committee of Sheba Medical Center (Approval No. 1117–14-SMC). Participants were recruited by their attending medical staff. The interviews were conducted by four palliative care nurses, and one social worker (two nurses which have an academic background in sexuality the other two received a dedicated workshop on end-of-life sexuality).

Participants were informed of their rights, including the freedom to withdraw from the study at any point without any impact on their medical care. It was clarified that all data would be anonymous and used solely for research purposes.

Interviews lasted from a few minutes to one hour, depending on the patient’s physical condition. Interviews were paused or shortened if the patient experienced distress. Even brief interviews often yielded significant insights due to the depth and authenticity of the participants’ responses.

## Data analysis

The interview data was analyzed using the Constant Comparative Analysis (CCA) method (Corbin and Strauss 2008). Each transcript was read in full, with preliminary notes and emergent themes recorded. Subsequently, interviews were broken down into meaningful segments or quotations, coded according to grounded theory principles.

Emerging categories were classified as either etic (literature-informed) or emic (data-driven). For instance, concerns related to self-image aligned with existing literature, whereas distress due to a perceived gap between emotional closeness and physical inability to maintain intimacy emerged from participant narratives.

The coding process continued until theoretical saturation was achieved. Each member of the research team conducted independent coding and then met in collaborative sessions to consolidate the thematic framework.

## Ensuring research validity

Two techniques were employed to enhance credibility:

1. Peer debriefing

Three external experts – two social workers and one nurse with expertise in palliative care – reviewed the results and provided feedback on interpretations (Lincoln and Guba 1985; Morse 2015).

1. Member checking

Due to the passing of all original participants, a terminal patient from the same hospice setting who was not part of the original study reviewed the synthesized findings. This patient confirmed that the interpretations accurately reflected their own experiences (Harvey 2015; Birt et al. 2016).

## Findings

### Variations in the experience of intimacy and sexuality during the dying process

The findings revealed a wide range of experiences regarding changes in intimacy and sexual functioning among terminal cancer patients. Based on participants’ narratives, three primary patterns emerged (See summary in Table 2). A separate section will focus on masturbation. Each quotation is accompanied by the participant number (P), gender (M/F), age, and cancer type (refer to the summary in Table 2).

**Table 2.** Variations in the experience of changes in intimacy and sexuality during the dying process

**No significant change in intimacy or sexuality**	1. Continuity of intimacy and sexuality
	2. Long-standing absence of sexual activity
**Significant change in intimacy and sexuality**	3. Diminished sexual functioning alongside maintained or enhanced intimacy

### Continuity of intimacy and sexuality

One of the more unexpected findings was the report by some participants of little to no significant change in their sexual and intimate lives during the dying process. These individuals described a continued need for and capacity to experience fulfilling intimacy, including sexual activity. Participants spoke of ongoing sexual desire, physical affection, such as hugging, caressing, and kissing and shared sexual pleasure. Beyond the physical aspects, some interviewees reported a deepening of emotional intimacy, characterized by shared humor, mutual emotional expression (e.g., crying together), and even a perceived improvement in their relational dynamics.

According to these participants, the ability to sustain and, in some cases, enhance intimacy was attributed to open and honest communication. This included candid discussions about the needs for physical affection, erotic touch, shared outings, and conversations regarding side effects, thoughts, and emotional experiences. Some reported a persistent sexual drive and mutual attraction, and did not experience common physical barriers such as vaginal dryness or pain during intercourse. Others did report changes in sexual desire or function but continued to engage in adapted forms of sexual activity. For these individuals, sexuality remained a source of emotional connection and a symbol of vitality and closeness, even in the face of terminal illness.

*Sex is a need, it is the finale of an entire story, it is an important necessity, and it is not just the sexual act. There is no change aside from being more careful because of the port. I do not have dryness or pain during penetration. When I look at my husband, I often think about sex with him too.* (P27, F, 37, Ovarian Cancer)

### Long-standing absence of sexual activity

Another subset of participants described an absence of sexual activity that predated their cancer diagnosis. These individuals often attributed the cessation of sexual relations to chronic illnesses (e.g., diabetes, cardiovascular disease, depression), side effects of medications (e.g., beta-blockers, antidepressants), or surgical interventions (e.g., prostatectomy). Some had adjusted to sleeping in separate beds or rooms, often due to conditions such as Parkinson’s disease, which disrupted shared sleep. While sexual activity was absent, expressions of affection and love – such as hugging and kissing – often persisted in non-sexual ways.

*I had my prostate operated on 14 years ago, which made me give up my sex life. There are hugs and kisses here and there. Not in a sexual way, but out of love…* (P15, M, 77, Prostate Cancer)

### Marked changes in intimacy and sexuality

In contrast, many participants reported significant changes in their sexual and intimate lives following their diagnosis and disease progression. A common theme was the avoidance of sexual activity, even among those who continued to experience sexual desire or erotic dreams. Some described a persistent longing for sexual connection, while others reported a complete absence of libido, fantasies, or interest in sexual pleasure. These changes were commonly attributed to physical symptoms – such as fatigue, pain, nausea, hypersensitivity to smells, or neuropathic sensations – as well as the presence of medical devices (e.g., catheters, ports) that hindered intimacy.

Despite these challenges, several participants reported continued emotional closeness and deepened relational bonds with their partners. In some cases, expressions of love and affection were enhanced through increased tenderness and caregiving.

*It used to be a different connection. A connection that is also… spiritual. Now I can’t, because of the odors. He knows. He understands me, but it’s tough for him and for me. Up until a month ago everything was as usual. I also wanted to feel myself, my body, my vitality recently. So, I asked him to touch me there. But I didn’t reach an… orgasm and that was okay. I just wanted intimacy. But he felt as if he let me down. He has never let me down. Sex for us is a very deep connection. He tries to show me affection in any way he can. He hugs, caresses… his presence – is all I need. I feel less out of place when he’s around.* (P20, F, 78, Leukemia)

### Masturbation and self-pleasure

Participants also reflected on changes in self-pleasure practices. While a minority reported no change in their masturbation habits, the majority who had previously engaged in self-pleasure described a cessation of the activity. This was commonly due to diminished sexual desire, a lack of physical sensation in the genital area, or an inability to become aroused or reach climax. Notably, some individuals reported that thoughts about sex and masturbation began to re-emerge after transitioning to palliative home care, although these thoughts did not necessarily lead to action.

*I don’t masturbate. I have no desire for sex. The battery charge is dropping.* (P31, M, 71, Pancreatic Cancer)

## B. Sources of distress related to changes in sexual functioning in the context of dying process

The interviewees expressed a variety of sources of distress they experienced following changes in their sexual functioning. Table 3 summarizes these sources, which are described in detail below.

**Table 3.** 6 D – Sources of distress experienced by interviewees due to changes in their sexuality

Source of distress		Explanation
B1. Personal source of distress	**D**eprivation of sexuality	Experience based on gap between patients’ need to have sex and their inability to achieve it
	**D**ual negative image	Negative self-image and negative body image which cause avoidance of creating new relationships and a feeling of loneliness.
	**D**eath thoughts	The discussion about sexuality, which is a complex topic, allowed for an open discussion on another complex topic, death.
B2. Marital source of distress	**D**ifficulties of the partner	Experience based on gap between partner’s need to have sex and patients’ inability to provide it.
	**D**istance and closeness	Experience is based on contradiction between the patients’ needs of physical contact with their beloved spouse and the need for distance at the same time. This is intensified due to side effects and the constant experience associated with nearing death.
	**D**o not experience concerns	No concerned by the changes in sexuality

## B1. Personal source of distress

### Distress stemming from sexual deprivation

A significant and novel theme that emerged from the interviews, including those conducted with participants in the terminal phase of illness (i.e., with a life expectancy of less than two weeks), was the emotional distress associated with sexual deprivation. This form of distress manifested in various ways: for some, it was rooted in grief over the loss of sexual desire; for others, it stemmed from the frustrating dissonance between a persistent need for sexual expression and the physical or psychological inability to engage in sexual activity.

For several participants, sexuality represented more than just physical intimacy; it was a central component of personal identity, a source of vitality, and a deeply integrated aspect of emotional well-being. The loss of this dimension of life was therefore experienced not only as a functional impairment but also as an existential rupture. One participant expressed a particularly poignant and emotionally charged account, emphasizing the profound psychological impact of medical interventions that resulted in chemical castration. His testimony reflects both the loss of sexual function and the symbolic erosion of identity and agency in the face of terminal illness:

*I used to be one of the most sexual people in Israel, no exaggeration. I remember that one of the hardest blows I got was the injections that castrated me. I used to love sex. Can I be rude? (preferably) I’d come home from work and check if my penis was hard. And suddenly they tell you: ‘You’re stirred!’ And you say: ‘I’ll be a castrato? I’ll go sing?’… It’s terrible, and because I’m also a member of the LGBQT community, I’m totally not marriage material. Today I’m totally sexual. I think they burnt it down completely… this injection kills any attraction… Say you were offered a tradeoff: we won’t inject you; you’ll die six months earlier but will have potency. Would you go for it? I would! And how much life would you give up on? Say they would give you five years to live with treatment, and without treatment – only six months of being sexually active. I would take six months. Take! Take! Take! Take! (P33, M, 50, Prostate Cancer)*

### Distress rooted in negative self-image and body image

For some interviewees – particularly those without a steady partner, the experience of advanced illness was compounded by a negative self-image and poor body image. These images contributed to emotional distress and social withdrawal. These individuals often described a reluctance to engage in romantic or sexual relationships, primarily due to concerns about physical appearance, visible signs of illness, and the anticipated reactions of potential partners. Feelings of unattractiveness, shame, and self-consciousness regarding their bodies were prominent, leading to a fear of rejection and a deepening sense of loneliness. This was especially pronounced in participants who found themselves navigating issues of intimacy while facing a life-limiting conditions.

One participant, a woman with metastatic lung cancer, articulated the emotional burden of these concerns, revealing how her self-perception and declining health created significant barriers to forming new relationships:

*I look terrible. I’m all bloated, fat and ugly. I have no desire to put on makeup and make myself pretty. I think I’m going to stay single. How can I date someone when I’m coughing and out of breath? And what would a man say if he knew I had metastatic lung cancer? I’m very sad. Partnership is important to me. Someone is trying to hit me on Facebook right now, but what will I tell him? (P28, F, 26, Lung Cancer)*

This narrative underscores the complex intersection of illness, identity, and intimacy, where the physical manifestations of disease can amplify emotional vulnerability and isolation.

### Distress stemming from death thoughts

The exploration of sexuality, a deeply personal and multifaceted subject, served as a gateway for participants to engage in conversations about another equally complex and often taboo topic – death. In the context of life-limiting illness, discussions surrounding sexuality did not occur in isolation but were inherently interwoven with broader existential concerns. As participants reflected on the transformations in their intimate lives, they were often simultaneously prompted to confront and articulate their thoughts and emotions regarding mortality, the process of dying, and the anticipated end of life.

The intimacy of discussing sexual identity, desires, and losses created a space of vulnerability and trust, enabling patients to address fears, hopes, and uncertainties about dying that might otherwise remain unspoken. In this way, sexuality functioned not only as a subject of inquiry but also as a relational and communicative bridge to the emotional landscape of terminal illness. Such dialogues highlight the potential for holistic palliative care approaches that recognize the interdependence of physical, emotional, sexual, and existential dimensions in end-of-life experiences.

*We evolved. We have reached the peak of our relationship. Now all the energy is channeled towards coping with the fear of death. There is a natural fear. We are educated to treat death as the end of everything. (P23, Female, 54, Ovarian Cancer)*

## B2. Marital source of distress

### Distress arising from the discrepancy between the partner’s sexual needs and the patient’s inability to fulfill them

Many participants reported experiencing emotional distress related to the perceived gap between their partners’ desire for sexual intimacy and their own physical inability to engage in sexual activity. This distress often manifested as a deep sense of guilt and concern over causing hardship or disappointment to their partners. Interviewees commonly express sadness, frustration, and disappointment with their inability to meet their partners’ sexual needs. For some, these feelings were compounded by a sense of shame and discomfort regarding changes in their bodies, which challenged their personal perceptions of masculinity or femininity. Others described fears that sexual activity might cause physical harm, either to themselves or their partners. Additionally, several participants expressed remorse for the emotional pain they may have caused in the past, as well as delayed recognition and appreciation for their partners’ emotional and physical support.

*Changes in erection bother me, though not a lot, because I find pleasure in other ways, but I feel like it affects my wife. She asked me to seek consultation, but I didn’t; I don’t know why. (P35, Male, 65, Sarcoma)*

### Distress resulting from the simultaneous need for closeness and the desire for distance

Several participants described emotional distress stemming from the complex and often contradictory experiences of needing physical closeness while simultaneously feeling a need to distance themselves due to physical symptoms such as nausea or heightened sensitivity to smells. This inner conflict created a deep sense of discomfort and anxiety, particularly due to the perceived impact on their partners. Participants reported a longing for intimacy, touch, and connection, juxtaposed against the physical aversions that made such contact difficult or impossible. The resulting tension often led to feelings of frustration, emotional pain, and fear of miscommunication within the relationship.

*Smells get to me. If he eats cold cuts, I can smell it and the nausea… I miss the sex and touch. He feels that I’m pushing him away. But it’s not true. I need him, but in a different way. When he’s not around me, I feel disconnected. Weak. He gives me – confidence. He thinks I don’t need him close to me anymore. That’s not true. I miss the touch, embrace, the kisses. But I can’t now, because of the smell. He understands me, but it’s tough for him and for me. Up until a month ago everything was normal. (P20, Female, 78, End-stage Leukemia)*

## B3. Absence of distress related to sexual dysfunction

In contrast, several interviewees reported no distress regarding their lack of sexual drive or ability. For some, this acceptance was attributed to habituation or the perception that reduced sexual activity was a natural part of aging or illness. Others emphasized that sex had become irrelevant in the face of their physical suffering or the urgent need to focus on survival.

*It does not bother me. I can’t now. My husband doesn’t feel the need himself either when he sees me suffering. He says: ‘You think I can do it when I see you suffering?’ He truly feels me. (P7, Female, 52, Pancreatic Cancer)*

### C. Interest in sexual counseling in the current situation

Approximately one-third of the interview participants, including some who were identified as terminally ill, expressed a desire to discuss issues related to sexuality and intimacy and to seek professional consultation. Several individuals expressed surprise at the idea of addressing sexuality in their current condition, noting that they had not considered the possibility that something could be done to improve their sexual functioning or even to open a dialogue about the changes they were experiencing. Some participants expressed a continued interest in sexual activity but refrained from engaging in it due to concerns about potential harm to themselves or their partners. For those who actively sought consultation and welcomed the opportunity, the motivation was often rooted in a desire to experience sexual intimacy again before the end of life, or in a wish to meet the sexual needs of their partners.

*I miss it a lot. I do not feel that my sexual desire has decreased, but I can’t because of a fracture in my back. I don’t know how to do it. No one asked us about it. I didn’t realize that sexual consultation could be received. I would be happy to receive consultation.* (P1, Female, 48, Bladder cancer)

In contrast, other interviewees described relationships in which sexual activity had ceased, and neither partner expressed a desire for consultation or intervention. Some participants found comfort in the emotional closeness and strengthened bonds with their partners, reporting no sense of sexual deprivation. Others voiced skepticism about the potential benefits of sexual counseling at this stage of illness, or indicated that their primary concerns were focused on the progression of their disease rather than on sexuality.

*I’m comfortable with not feeling sexual desire now and don’t seek to change it. I’m happy like this and am very pleased with the relationship and the closeness between us.* (P2, Male, Lung cancer)

### D. The role of healthcare teams in initiating conversations about sexuality

A question that remains underexplored in the existing literature is how terminally ill patients perceive the role of healthcare professionals in initiating conversations about sexuality and intimacy within the clinical setting. In this study, approximately 80% of participants expressed a strong belief that it is the responsibility of the care team to introduce the topic, rather than waiting for patients to do so. Participants emphasized that once the topic is raised by the professional team, patients can then decide whether they feel comfortable engaging in the discussion or receiving related consultation.

Several participants noted that they might be interested in continuing sexual activity during the final stages of life, particularly those for whom sexuality held significance prior to their illness. Some individuals expressed a desire to ask questions but refrained from doing so due to feelings of embarrassment or shame. Others highlighted that patients are often unaware that sexuality is a topic that can be discussed with medical professionals, or that specialized consultation is available. Several interviewees also underscored the importance of discussing sexuality at this stage of life as a means of addressing emotional or existential concerns, suggesting that such conversations may play a role in psychological or spiritual *coping.*

*If it was important for someone during their lifetime, it would be important till the end. It’s important the team asks. Consultation is important because it’s better to have a good, vibrant, and short life than a gray and long one. (P6, Female, 59)*

## Discussion

This study explored the multifaceted experiences of terminal cancer patients in relation to their sexuality – an area that remains underrepresented in palliative care research. Key themes that emerged include the types of distress patients face due to changes in sexuality, perceptions surrounding masturbation, the role of the palliative care team in addressing sexual concerns, and the expressed need for sexual consultation during the dying process. In addition, the interviews created a unique space in which deeply personal conversations about death and dying could unfold through the lens of intimacy and sexuality.

### Are dying patients interested in sex?

Contrary to prevailing assumptions in palliative care – namely, that patients nearing death are preoccupied with physical symptoms and therefore disinterested in sexuality (Cagle and Bolte 2009; Leung et al. 2016; Benoot et al. 2018; Wallach et al. 2023) – the present study found that intimacy and sexuality remain meaningful concerns for a significant number of terminally ill individuals. Many participants emphasized a desire for sexual consultation, with approximately one-third expressing an explicit interest in receiving support for sexual issues even during the final stages of life.

The testimonies of the interviewees powerfully illustrate how sexual function can remain deeply meaningful even in the final stages of life. One of the participants’ willingness to trade significant time off his life expectancy for the chance to retain sexual capacity underscores the centrality of sexuality to his quality of life and identity. Moreover, the experience of the interviewees highlights how treatment decisions, while medically justified, may carry significant emotional and psychosocial consequences, particularly for individuals whose self-concept is closely linked to their sexual agency and expression.

Notably, a recurrent theme among participants was the expectation that healthcare providers, particularly those on palliative care teams, should initiate conversations about sexuality. This expectation stems from a general lack of awareness among patients regarding the legitimacy and feasibility of addressing these issues within the clinical setting. This finding suggests a critical need for palliative care teams to proactively introduce discussions about sexuality, thereby validating patients’ experiences and needs at the end of life.

### Distress related to sexual changes

The findings underscore the complex and deeply personal nature of sexual distress experienced by individuals facing terminal illness. This study illuminates the multifaceted sources of distress that result from changes in sexuality at the end of life.

A primary theme that emerged was the emotional suffering tied to sexual deprivation, which extended far beyond physical function and touched on core aspects of identity and personhood. Participants expressed grief not only for the physical loss of sexual capability but also for what this loss represented in terms of vitality, self-expression, and autonomy.

In addition to the loss of sexual function, participants described significant distress rooted in negative self-image and body image. These concerns were often exacerbated by visible symptoms of illness, which participants feared would elicit rejection or pity from potential partners. These findings align with existing literature on the stigmatizing effects of visible illness on intimacy (Sanchez and Kiefer 2007; Elmerstig et al. 2016) and suggest that body image may be an underappreciated contributor to end-of-life suffering.

Another salient theme was the way that conversations about sexual changes often served as an entry point to discussing death itself. In many cases, reflections on sexual loss prompted patients to articulate their fears, values, and emotional responses to the dying process. This speaks to the deeply interconnected nature of sexuality and existential awareness and reinforces calls for holistic models of palliative care that address not only physical symptoms but also the emotional, relational, and spiritual needs of patients.

The study also identified sources of marital distress, particularly the emotional burden associated with an inability to meet a partner’s sexual needs. Feelings of guilt, shame, and inadequacy were recurrent themes. For many, there was a palpable tension between the desire for emotional and physical closeness and the simultaneous need for distance due to illness-related symptoms. This ambivalence mirrors findings from dyadic studies on caregiving and intimacy at the end of life, which have shown that physical closeness can sometimes trigger discomfort or even revulsion in patients, despite emotional longing (Badr and Acitelli 2005).

Interestingly, participants often expressed concern not only for their own suffering but also for the emotional needs of their partners, highlighting the relational dimensions of distress that are often overlooked in individual-centered palliative care models. These findings argue for a more systemic approach to end-of-life support, one that considers the patient in the context of their intimate relationships.

The study also identified sources of marital distress, particularly the emotional burden associated with an inability to meet a partner’s sexual needs. Feelings of guilt, shame, and inadequacy were recurrent themes, often compounded by concerns about altering the dynamics of long-standing relationships. For many, there was a palpable tension between the desire for emotional and physical closeness and the simultaneous need for distance due to illness-related symptoms. This ambivalence mirrors findings from dyadic studies on caregiving and intimacy at the end of life, which have shown that physical closeness can sometimes trigger discomfort or even revulsion in patients, despite emotional longing (Badr and Acitelli 2005).

In contrast to the above, several participants reported no distress related to sexual dysfunction. This subset of participants described their sexual changes as a natural and expected consequence of terminal illness, aligning with findings from Hendrickx et al. (2016). For these individuals, acceptance of bodily transformation and altered sexual norms facilitated a greater sense of peace, suggesting that psychological adjustment plays a protective role against distress. This observation supports existing literature indicating that self-acceptance and cognitive reframing can mitigate suffering in the face of diminishing physical capacities (Elmerstig et al. 2016). This points to significant variability in how sexual loss is experienced and contextualized, echoing prior findings that underscore the importance of individual differences in coping, values, and cultural narratives around sexuality and illness (Reisfield and Wilson 2004). Importantly, these narratives suggest that not all patients experience sexual loss as distress, and that distress may hinge more on meaning than on functionality

### Understanding variability in sexual interest among the dying

A striking finding in this study was the heterogeneity in participants’ interest in sexuality near the end of life. For some, sexual activity persisted as a vital, even existential, expression of life. This phenomenon echoes classical and contemporary psychological theories that frame sexuality as a life-affirming force capable of mitigating death anxiety (Freud, as cited in Riviere, Freud 1962; Schover 2019; Shaver and Mikulincer 2012). In this context, sexuality functions not merely as a pursuit of physical pleasure but as a reaffirmation of one’s humanity and vitality amidst terminal decline.

Conversely, participants who expressed no interest in sexual activity may be understood through an existentialist framework, particularly that of Kierkegaard (2004a, 2004b). According to Kierkegaard, individuals progress through stages of aesthetic, ethical, and spiritual existence. While those in the aesthetic or ethical stages may view the loss of sexuality as a personal or relational failure, those situated in the spiritual stage may transcend physical desires in pursuit of spiritual fulfillment. Søltoft (2016) argues that such spiritual development allows individuals to relinquish temporal concerns, including sexual identity, as they turn toward a transcendent understanding of self and existence. This philosophical lens may account for the peace and lack of distress reported by certain participants.

### Sexuality, death, and anticipatory grief

Another significant insight from this study is the intersection between sexuality and anticipatory grief. Participants frequently transitioned from discussing their sexual identity to reflecting on their impending death. This suggests that the decline of sexual functioning can serve as a symbolic precursor to the broader losses associated with dying. Anticipatory grief, defined as the emotional response to foreseen loss, includes mourning the gradual erosion of one’s lifestyle, autonomy, roles, and relationships (Gotoh et al. 2025). The loss of sexual intimacy may thus represent a critical aspect of this pre-death mourning process.

Participants described a range of emotional reactions to these changes – grief, frustration, guilt, and sorrow – often related to their perceived inability to maintain intimate connections or fulfill partner expectations. These reactions are consistent with previous findings linking anticipatory grief to role loss and identity dissolution (Hottensen 2010; Shore et al. 2016; Patinadan et al. 2022). Conversely, other participants expressed increased intimacy with their partners despite changes in sexual behavior. This shift suggests that emotional closeness may persist or even deepen when physical intimacy wanes. Indeed, acceptance has been identified as a key psychological predictor that mitigates the distress of anticipatory grief and promotes well-being at the end of life (Davis et al. 2017).

### Role of healthcare professionals

A key finding is that 80% of participants felt it was the responsibility of the healthcare team to initiate conversations about sexuality. Patients often hesitate to bring up such topics due to embarrassment, stigma, or assumptions that the subject is inappropriate in medical contexts. This highlights a crucial role for providers: legitimizing and creating space for these discussions.

Proactive engagement may validate patients’ experiences and offer them access to resources that enhance quality of life. It also provides an opportunity to address misconceptions, such as the belief that sexual activity is impossible or inappropriate during the dying process.

Considering these findings, the study strongly advocates that sexuality, and intimacy should not be marginalized in palliative care. Instead, they should be integrated into psychosocial support frameworks to ensure holistic, patient-centered care.

## Conclusion, implications, and recommendations

This study challenges prevailing assumptions that intimacy and sexuality become irrelevant in the context of terminal illness. On the contrary, the findings demonstrate that for many individuals nearing the end of life, sexuality continues to serve as a critical dimension of identity, connection, vitality, and existential meaning. The distress expressed by some participants over diminished sexual functioning underscores the necessity of integrating compassionate, patient-centered dialogue on this topic into end-of-life care. Notably, a substantial proportion of participants expressed openness to discussing sexuality and a willingness to engage in professional counseling, further emphasizing the importance of addressing this domain. Moreover, the expectation among participants that healthcare providers should initiate these conversations points to a clear responsibility within clinical practice – particularly given patients’ frequent reluctance to broach the topic themselves due to stigma, discomfort, or lack of awareness.

One of the most significant contributions of this study is the revelation that a substantial number of terminally ill patients welcome the opportunity to discuss sexuality and would be open to professional counseling. Additionally, most participants felt that it was the responsibility of the healthcare team to initiate this discussion, especially given that many patients may feel embarrassed, unaware of the option, or reluctant to raise the issue themselves.

These findings support a more holistic approach to palliative care, one that recognizes sexuality not merely in biomedical or psychosexual terms, but as a meaningful component of emotional, relational, and spiritual well-being. A comprehensive model of care that affirms patients’ right to intimacy can play a vital role in preserving dignity and enhancing quality of life at the end of life.

Sexuality at the end of life should not be viewed solely through a biomedical or psychosexual lens, but as an integral aspect of a patient’s emotional, relational, and even spiritual experience. Palliative care teams must adopt a holistic view that validates patients’ right to intimacy and fosters dignity and meaning at life’s end.

### Practical implications for palliative care staff

To integrate sexuality more fully into end-of-life care, we propose the following ABC framework:

- ***Ask***

Proactively ask terminal patients about their interest in discussing intimacy and sexuality, without making assumptions. Respect their boundaries and offer space for individual preferences.

- ***Bridge***

Act as a bridge between the patient and specialized professionals – such as urologists, gynecologists, psychologists, sex therapists, or spiritual counselors – who can offer tailored support.

- ***Combine***

Address sexual well-being through an integrative approach that blends medical, psychosocial, and spiritual strategies, tailored to the patient’s unique context.

This approach does not necessarily aim to restore previous sexual function, but rather to help patients rediscover new forms of intimacy and fulfillment, or to say farewell to that dimension of life with a sense of peace and closure.

### PASSION model: Recommendations for multilevel intervention

The findings of this study also support the implementation of the **PASSION** model – a multidimensional framework that includes recommendations for action at the personal, organizational, and systemic levels:

**Table:** 

Dimension	Explanation
**P** – *Personal responsibility*	Encourage every member of the palliative care team to examine their own beliefs and barriers related to discussing sexuality. Staff should also participate in formal training.
**A** – *Alive*	Professionals should adopt the perspective that patients are fully alive – emotionally, physically, and sexually – even as they approach death. As Dame Cicely Saunders said: *“You matter because you are you, and you matter to the end of your life.”*
**S** – *Signification*	Support patients in finding meaning in their experiences of intimacy and sexuality during their final days.
**S** – *Search*	Promote further research on (a) the impact of training of healthcare providers’ attitudes, (b) the outcomes of interventions targeting intimacy at the end of life, (c) the development of culturally sensitive assessment tools, and (d) the phenomenon of anticipatory grief related to sexual loss.
**I**–*Institutional responsibility*	Establish institutional protocols such as staff training, inclusion of sexual health in intake forms, and the appointment of designated sexual health coordinators within palliative teams.
**O** – *Offer*	Encourage care providers to offer – not impose – a conversation about sexuality, thereby normalizing the topic and reducing stigma.
**N** – *Networking*	Build partnerships with professionals across disciplines to ensure comprehensive care. Suggested collaborators include sex therapists, nurses, oncologists, spiritual care providers, and social workers.

## Strengths and limitations

This study’s primary strength lies in its focus on a neglected yet meaningful topic: sexuality at the end of life. By conducting in-depth interviews with terminal patients, it captured voices and needs that are often unaddressed in medical literature. The inclusion of patients from both hospital and home hospice settings provided a broader perspective across different care environments.

However, several limitations must be acknowledged. First, data collected by different interviewers with varying clinical experience and communication styles, which may have influenced the depth and tone of the interviews. Second, all participants were recruited from the center of Israel, thus not fully representing the cultural diversity existing in the state (cultural, religion and ethnicity). Third, due to physical fatigue, some interviews were cut short, which may have restricted the richness of data from certain individuals.

Despite these limitations, the insights gained are valuable and provide a foundation for further exploration of sexual and emotional needs among the terminally ill. Future research would benefit from broader recruitment across diverse cultural and religious populations, and the use of standardized tools to assess sexual well-being in palliative care contexts.

## Sexual Function – Interview Guide

**Participant Questionnaire**

**Participant No.:** ___________ **Date**: ___________

It is widely recognized that many individuals in your situation experience changes in their intimate lives. While some find these changes distressing and wish to discuss or address them, others may feel less inclined to engage in such conversations.

I will be asking you questions related to your intimate life. You are free to choose which questions you feel comfortable answering. We will begin with a few questions regarding personal background.

**Demographic Information**

A. Date of Birth: _______________________

B. Country of Birth: ____________________

C. If immigrated, when __________________

D. Ethnic Background:

1. Israeli 2. Sephardic/North African 3. European 4. Mixed

E. Sexual Orientation:

1. Heterosexual 2. Homosexual 3. Bisexual

F. Marital Status:

1. Married 2. Widowed 3. Divorced 4. Single 5. Separated 6. Other: ___________

G. If you have a partner: Type of Relationship:

1. First marriage 2. Second marriage 3. Committed relationship 4. Domestic partnership

H. Duration of Relationship (years): __________ Do you live together? 1. Yes 2. No

I. Number of Children: __________ Number of Grandchildren: __________

J. Education Level:

1. Elementary 2. High School 3. Academic Degree

K. Profession: ____________________________

L. Past and Current Occupation: _______________________

M. How would you describe your financial situation (in Israel SES is referred as financial situation)

1. Poor 2. Average 3. Good 4. Excellent

N. Religious Affiliation or Level of Observance:

1. Religious 2. Secular 3. Traditional 4. Other: __________

O. Type of Cancer: ___________________________

P. Treatments Received:

1. Chemotherapy 2. Radiation 3. Hormonal Therapy 4. Surgery 5. Biological Therapy

Additional Details: ___________________________________________

Q. Other Diagnosed Medical Conditions: _________________________

R. Other Medications: __________________________________________

S. Level of Functional Independence:

1. Independent 2. Partially Dependent 3. Dependent on others for most activities

Interview Questions

1. What does a fulfilling intimate life mean for you? (e.g., physical touch, listening, caressing, sexual activity, open communication).

2. How would you describe your intimate life before your illness?

3. Many individuals report experiencing changes in intimacy during illness. Have you noticed any such changes? If so, can you describe them? Were any of these changes positive? If yes, in what way? Were any negative? Please elaborate.

4. How do you feel about the changes you’ve described? To what extent did these changes cause you distress if at all?

5. What aspects of your intimate life bring you satisfaction today? What do you miss the most?

6. With your permission, I would like to focus specifically on sexual behaviors. Many people report changes in their sexual habits due to illness or treatment. Have your sexual habits changed? If so, how?

7. Are there things that still give you pleasure or arouse you? Have you experienced sexual thoughts or fantasies over the past month? Can you describe when and in what context these occur? How often? Do you enjoy sexually themed books, films, or art? Have you had erotic dreams? If so, do you remember them, and how frequently do they occur? Can you recall a specific moment and how it made you feel?

8. How would you currently describe your level of satisfaction or enjoyment in your sexual life? (e.g., very enjoyable, OK, not enjoyable at all)

9. Are there any issues that bother you? (e.g., decreased libido, difficulty reaching orgasm, erectile dysfunction, lack of ejaculation)

10. Do you remember the last time you had sexual intercourse? How frequent does it occur?

11. Some people in your situation engage in self-pleasure occasionally, while others do not. Is this something you do?

12. Some individuals seek support or counseling regarding these issues; would you be interested talking about it?

13. Is there anything else regarding your intimate or sexual life that you would like to share or tell me about??

**Thank you very much for your openness and cooperation.**
